# Enzyme-Treated *Zizania latifolia* Ethanol Extract Protects from UVA Irradiation-Induced Wrinkle Formation via Inhibition of Lysosome Exocytosis and Reactive Oxygen Species Generation

**DOI:** 10.3390/antiox9100912

**Published:** 2020-09-24

**Authors:** Mirae An, Hyungkeun Kim, Joo-Myung Moon, Hyun-Soo Ko, Paul Clayton, Young-Hee Lim

**Affiliations:** 1 Department of Public Health Science (BK21 PLUS Program), Graduate School, Korea University, Seoul 02841, Korea; mran318@naver.com; 2 Department of Oral Biology, Oral Cancer Research Institute (BK21 PLUS Program), Yonsei University College of Dentistry, Seoul 03722, Korea; khg165@gmail.com; 3 Department of Applied Life Science, Graduated School, Yonsei University, Seoul 03722, Korea; 4 BTC Corporation, Ansan, Gyeonggi-do 15588, Korea; mhjj1919@btcbio.com; 5 Department of Integrated Biomedical and Life Sciences, Graduate School, Korea University, Seoul 02841, Korea; rhgustn0303@naver.com; 6 Institute of Food, Brain and Behaviour, Beaver House, 23-38 Hyde Bridge Street, Oxford OX1 2EP, UK; Paulrclayton@gmail.com; 7 Department of Laboratory Medicine, Korea University Guro Hospital, Seoul 08308, Korea

**Keywords:** antiwrinkle, lysosomal shedding, matrix metalloproteinases, ultraviolet A, *Zizania latifolia*

## Abstract

Ultraviolet A (UVA) is a risk factor for photoaging and wrinkle formation. *Zizania latifolia* is an herbaceous perennial plant. It contains many bioactive compounds such as tricin that show antioxidative and anti-inflammatory effects. The aim of this study was to investigate the antiwrinkle effect of a mixture of hydrolytic enzyme (cellulase, hemicellulase and pectinase)-treated *Z. latifolia* extract (ZLE) and tricin on UVA-irradiated human dermal fibroblasts (HDFs) and SKH-1 hairless mice. Treatment of UVA-irradiated HDF cells with ZLE and tricin significantly decreased UVA induced-plasma membrane rupture, generation of ROS, expression levels of total and secreted lysosomal associated membrane protein (LAMP-1), cathepsin B and metalloproteinases (MMPs) and inhibited NF-κB activation. In the animal study, UVA-damaged epidermal and dermal tissues were repaired by the ZLE and tricin treatments. Administration of ZLE or tricin to UVA-irradiated animals recovered skin surface moisture and collagen fiber in dermal tissue. Treatment of ZLE or tricin decreased wrinkle formation, secretion of MMPs and expression levels of vascular endothelial growth factor (VEGF) and cathepsin B, and increased the expression level of collagen-1 in UVA-irradiated animals. Overall, the ZLE and tricin treatments decreased the skin damage induced by UVA irradiation via inhibition of lysosomal exocytosis and ROS generation. Therefore, ZLE and tricin are promising as antiwrinkle and antiphotoaging agents.

## 1. Introduction

Ultraviolet (UV) radiation of the skin is known as a primary risk factor for environmentally influenced skin disorders. Homeostatic imbalances of the skin caused by UV radiation may result in winkles, rashes, skin cancers, lack of moisture and immune-related disorders [1]. UV radiation is divided into three bands based on the wavelengths: UVA (315–400 nm), UVB (280–315 nm) and UVC (<208 nm). UVA and UVB radiation are the main risk factors for premature skin aging and skin cancer, respectively. By comparison, UVC is of less concern than UVA and UVB because of its absorption into the ozone layer. UVA penetrates deeper into the skin to the epidermis and dermis than UVB and UVC because of its longer wavelength. In everyday life, people are easily exposed to UVA, which constantly induces skin homeostasis imbalance [2,3].

Reactive oxygen species (ROS) are reactive chemical species containing radical and nonradical oxygen. Cellular ROS are generated endogenously during mitochondrial oxidative phosphorylation and play roles in cell signaling [4]. In skin physiology, environmental stimuli such as UV rays, exposure to cigarette smoke and fine dust contribute to the generation of ROS, which in turn induces intrinsic damage and photoaging in human skin [5]. UV-induced ROS serve as central mediators in regulating the proteolytic degradation of the skin extracellular matrix (ECM) by up-regulation of matrix metalloproteinase (MMP) expression through the nuclear factor-κB (NF-κB) signaling pathway [6,7].

Lysosomes are acidic organelles that contain many proteolytic enzymes. Lysosomes play an important role in cutaneous physiology such as keratinization and pigmentation and in cutaneous pathophysiology such as inflammation and neoplasia [8]. Melanocytes are important to protect skin against UV radiation. UVA induces lysosomal exocytosis in melanocytes followed by shedding of extracellular vehicles that are transferred to keratinocytes [9], which suggests that UVA damages melanocytes by lysosomal exocytosis that results in the destruction of the UV protection system in the skin. Lysosomal disruption in the cytosolic compartment may cause cell damage directly and consequently induce skin cell death [10]. However, it is controversial whether lysosomal exocytosis affects skin health by hindering plasma membrane repair and degrading the ECM. In the repair system, lysosomal exocytosis acts as a sealing mediator by localizing acid sphingomyelinase at the plasma membrane where it participates in endosome formation and plasma membrane repair [9,11]. However, several proteases such as the hydrolytic enzymes from the aspartic, cysteine and serine protease families are secreted into the extracellular space from the lysosome by ECM degradation [12]. Degradation of the ECM may cause an inflammatory microenvironment and result in inadequate ECM turnover and remodeling with wrinkle formation or by aggravating the moisture content in the skin [13].

*Zizania latifolia* Turcz (Poaceae, known as wild rice) is an herbaceous perennial plant native to East Asia. As a food, its stem and grain are used as a vegetable source. The leaves of *Z. latifolia* show various beneficial effects on the improvement of metabolic and cardiac diseases and hypertension [14,15,16,17]. *Z. latifolia* also has protective effects on UVB induced-wrinkle formation and skin moisture content as well as antioxidant activity through regulation of the mitogen-activated protein kinase (MAPK) signaling pathway [18,19]. The leaves of *Z. latifolia* contain proteins, minerals, vitamins and many bioactive compounds. In particular, the flavonoid tricin found in the leaves of *Z. latifolia* was shown to improve the cutaneous system by regulating antioxidative and anti-inflammatory signaling pathways [20,21]. The concentration of tricin increased in an ethanol extract of the aerial parts of *Z. latifolia* after they were treated with a mixture of hydrolytic enzymes (cellulase, hemicellulase and pectinase) [19]. In this study, we investigated the protective effects of enzyme-treated *Z. latifolia* extract (ZLE) and tricin on skin aging by UVA radiation through inhibition of lysosomal shedding and increased antioxidant activity. This study was proved a new function of antioxidants that can prevent UVA irradiation induced-wrinkle formation via inhibition of lysosomal shedding.

## 2. Materials and Methods

### 2.1. Chemicals

High performance liquid chromatography (HPLC)-grade acetonitrile, methanol and water were purchased from Merck (Darmstadt, Germany). Phosphoric acid, 3-(4,5-dimethylthiazol-2-yl)-2,5-diphenyltetrazolium bromide (MTT) and dimethyl sulfoxide (DMSO) were obtained from Sigma-Aldrich (St. Louis, MO, USA). Tricin was purchased from Chromadex (Irvine, CA, USA).

### 2.2. High Performance Liquid Chromatography (HPLC)

**Figure 1:**
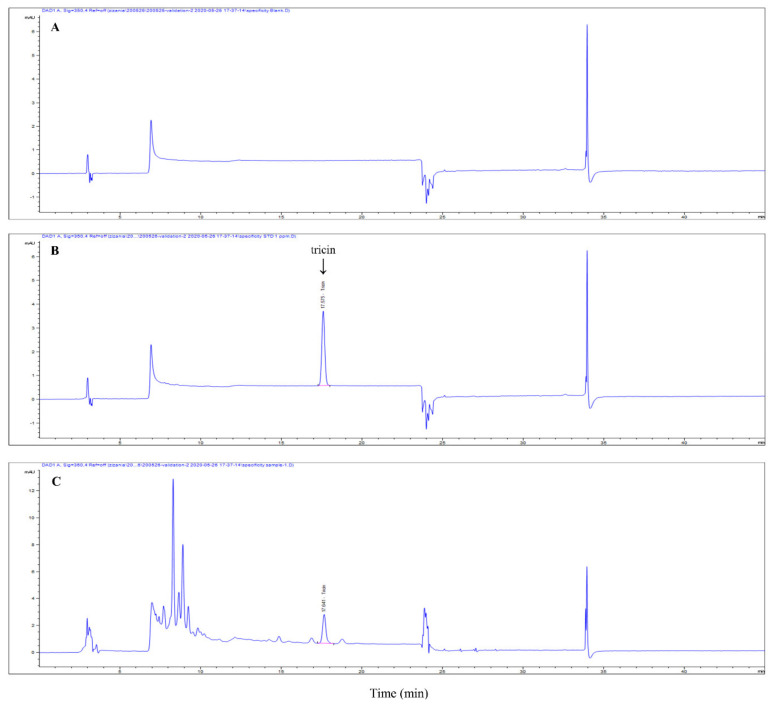
HPLC chromatograms of (**A**) blank, (**B**) tricin standard and (**C**) enzyme-treated *Z. latifolia* extract (ZLE).

The aerial parts of *Z. latifolia* were purchased from Pureunsan Agricultural Association Corporation (Pocheon, Korea) and ZLE, prepared according to a previously described method [18], was provided from the BTC Corporation (Sangnok-gu, Ansan, Korea). The concentration of tricin in ZLE was determined by HPLC using an Agilent Infinity 1260 system (Agilent Technologies, Palo Alto, CA, USA), which was measured in our previous study [19]. The separation of tricin was conducted at 30 °C using a SUPELCO Discovery^®^ C18 column (4.6 × 250 mm, 5 μm, Merck KGaA). The mobile phase composition was as follows: A, 0.15% phosphoric acid in water; B, methanol. The gradient elution conditions were as follows: 0–3 min, 20% B; 3–8 min, 20%–50% B; 8–20 min, 50%–55% B; 20–30 min, 55%–85% B; 30–45 min, 85%–20% B. The flow rate was 1.0 mL/min, and the injection volume was 10 μL. The chromatograms were obtained at 340 nm (Figure 1). The concentration of tricin was 0.9 mg/g ZLE.

### 2.3. Cell Viability

Human dermal fibroblasts (HDFs) were purchased from the ATCC (Manassas, VA, USA). Cells were cultured in fibroblast basal medium supplemented with fibroblast growth kit-low serum (ATCC) and a 0.5% penicillin-streptomycin-L-glutamine mixture (Lonza, Walkersville, MD, USA) that was used as a complete medium, in a 5% CO_2_ atmosphere at 37 °C. After the cells reached around 80% confluence, cells were collected by treatment with 0.05% trypsin-EDTA (Thermo Fisher Scientific, Grand Island, NY, USA). HDFs (1 × 10^4^ cells/well) were seeded in a 96-well plate (SPL, Pocheon, Korea) and cultured in the complete medium. After 24 h, the cells were treated with ZLE (0.5 and 1 μg/mL) or tricin (1 ng/mL) in the complete medium for 24 h, and then, the medium was changed to phosphate buffered saline (PBS) containing 0.9 mM CaCl_2_. Then, the cells were irradiated with ultraviolet A (UVA) irradiation (20 J/cm^2^) using a BIO-LINK^®^ crosslinker (BLX, Vilber Lourmat, France) after ZLE or tricin-treatment. To measure cytotoxicity, the cells were treated with 20 μL MTT (5 mg/mL in PBS) at 37 °C for 4 h. DMSO (200 μL) was added to each well to dissolve the formazan crystals, and the cells were incubated for an additional 30 min at 37 °C. Then, the absorbance was determined at 570 nm using a microplate reader (FLUOstar Omega, BMG LABTECH, Ortenberg, Germany).

### 2.4. Determination of Intracellular ROS Generation

Generation of intracellular ROS was measured using a 6-corboxy-2′,7′-dichlorodihydrofluorescein diacetate (DCF-DA) fluorescent probe (Invitrogen, Waltham, MA, USA). The cells were treated with ZLE (0.5 and 1 μg/mL) or tricin (1 ng/mL) in the complete medium for 24 h. DCF-DA in sterile DMSO was prepared just before irradiating the HDFs. The HDFs were washed with PBS followed by irradiation with 20 J/cm^2^ of UVA, and then, DCF-DA was added at a final concentration of 1 μM in PBS. The cells were incubated at 37 °C in 5% CO_2_ for 30 min in the dark and then were washed with PBS three times. The cells were observed using a microplate reader (FLUOstar Omega) with excitation and emission spectra of 490 and 525 nm, respectively.

### 2.5. Calcium Influx Assay

The cytosolic Ca^2+^ concentration was evaluated using the fluorescent Ca^2+^ indicator Fluo-4/AM (Thermo Fisher Scientific). Briefly, 1 × 10^4^ cells/well of HDFs were seeded in clear flat-bottom black 96-well culture plates with the complete medium overnight. The cells were treated with ZLE or tricin at the indicated concentrations in the complete medium for 24 h. Then, the medium was changed to Hanks Balanced Salt Solution (HBSS, Thermo Fisher Scientific) supplemented with 2 μM Fluo-4/AM and incubated for 30 min at 37 °C. The cells were washed with HBSS three times, and 200 μL PBS containing 0.9 mM CaCl_2_ was added. After treatment with 20 J/cm^2^ UVA irradiation, the cytosolic Ca^2+^ concentration was then evaluated using a microplate reader (FLUOstar Omega). The excitation and emission wavelengths were 490 nm and 525 nm, respectively. All measurements were performed in triplicate at least three times independently.

### 2.6. Gelatin Zymography

The protein concentration in the cultured media was determined using a Pierce BCA Protein Assay Kit (Thermo Fisher Scientific). The protein (20 μg) was applied to an 8% sodium dodecyl sulfate (SDS)-polyacrylamide gel containing 0.2% (*w/v*) gelatin. After electrophoresis, the gel was washed twice with 2.5% Triton X-100 for 1 h at room temperature and incubated in a buffer containing 50 mM Tris-HCl (pH 7.4), 0.02% NaN_3_, 10 mM CaCl_2_ and 150 mM NaCl for 24 h at 37 °C under a humidified atmosphere. The gel was stained with a solution of 0.1% Coomassie Brilliant Blue R-250 (Fluka Chemie AG, Ne-Ulm, Switzerland). The gelatinolytic activities of matrix metalloproteinases (MMPs) were detected as transparent bands against the background of the Coomassie Brilliant Blue-stained gelatin and quantified using ImageJ.

### 2.7. Immunofluorescence

HDF cells (1 × 10^4^ cells/well) were seeded in a chamber slide and incubated in the complete medium overnight. Then, the cells were treated with ZLE or tricin at the indicated concentrations in the complete medium for 24 h and then irradiated with 20 J/cm^2^ UVA. The cells were fixed with 4% paraformaldehyde (*w/v*) at 4 °C overnight and permeabilized with 0.1% Triton X-100 for 10 min. After blocking with 2% goat serum in PBS, the cells were incubated with anti-NF-κB (1:500, Thermo Fisher Scientific) in 2% goat serum in PBS at 4 °C overnight. After washing, the cells were incubated with Alexa Fluor 488 goat antirabbit IgG (Invitrogen) for 1 h at room temperature. The slide was mounted using Vectashield mounting medium with DAPI (Vector Laboratories, Burlingame, CA, USA). The images were obtained using a Zeiss LSM 800 confocal microscope (Carl Zeiss, Oberkochen, Germany).

### 2.8. Experimental Animals and UVA Irradiation

Six-week-old female SKH-1 hairless mice were purchased from Orient Bio (Seongnam, Korea). The mice were housed at 22 ± 1 °C, relative humidity of 50% ± 5% and intensity of illumination of 200 ± 50 lux under a 12 h light–dark cycle. All experimental protocols were carried out following an institutionally approved protocol from the Korea University institutional animal care and use committee (Approval No. KUIACUC-2019-0030). All mice were fed a rodent diet (No. 5053, LABDIET, St. Louis, MO, USA) and had free access to food and water. After a week of acclimation, mice were randomly divided into six groups (n = 10/group). Group 1 (the normal control: NC) was treated with vehicle (PBS) without UVA irradiation; Group 2 (the UVA-irradiated control: UC) was treated with vehicle and UVA irradiation; Group 3 (the positive control: AA) was treated with ascorbic acid (50 mg/kg/day) and UVA irradiation; Group 4 (ZLE100) was treated with ZLE (100 mg/kg/day) and UVA irradiation; Group 5 (ZLE200) was treated with ZLE (200 mg/kg/day) and UVA irradiation; Group 6 (Tri) was treated with tricin (0.5 mg/kg/day), an active component of ZLE, and UVA irradiation. ZLE, ascorbic acid and tricin were orally administrated to the indicated groups daily for 10 weeks, while the normal control and UVA-irradiated control were orally administrated PBS. Body weight was measured every three weeks.

The mice were irradiated with UVA for 10 weeks. The intensity of UVA was 100 J/cm^2^/week, and irradiation was performed in a manufactured acrylic cage with UVA lamps (15 W, emission intensity 356 nm, distance 18 cm). UV power was measured with an UV radiometer (UV-340A, Lutron electronic, Lehigh, PA, USA). The mice were irradiated five times a week, which was carried out from Monday to Friday for 2 h per day.

### 2.9. Serum Biochemical Analysis

The mice were fasted for 24 h before sacrifice under isoflurane anesthesia. Blood was collected by cardiac puncture and centrifuged for 10 min at 3000× *g* to separate the serum. Serum was used to measure glutamic oxaloacetic transaminase (GOT) and glutamic pyruvic transaminase (GPT) using FUJI DRY-CHEM slides (FUJIFILM Co. Tokyo, Japan).

### 2.10. Analysis of Dorsal Skin Surface

The dorsal skins of the mice at 5, 8 and 10 weeks after irradiation with UVA were analyzed using a multiskin test center MC750 (Courage+Khazaka Electronic GmbH, Cologne, Germany) to measure moisture content, transepidermal water loss (TEWL) and the dermal erythema index. Room temperature and relative humidity were maintained at 22 ± 1 °C and 50% ± 5%, respectively, during the measurements. The dorsal skins of mice were also replicated using a skin replica kit (repliflo cartridge kit, Medico, Ansan, Korea) after placing the mice under anesthesia using isoflurane at 5, 8 and 10 weeks after irradiating with UVA. Mean wrinkle depth, mean wrinkle length and mean form factor were analyzed using a Visioline VL650 (Courage and Khaxaka Electronic GmbH).

### 2.11. Histological Analysis

The dorsal skins were fixed with 10% formalin immediately after collection, gradually dehydrated in 80%, 90% and 100% ethanol and cleaned with xylene. Next, the tissues were embedded in paraffin, and hematoxylin and eosin (H&E) staining was carried out. Masson’s trichrome staining was performed for the detection of collagen fibers in the skin tissue. The paraffin-embedded tissues were placed in a 5% iron alum solution for 30 min at 56 °C and then stained with Weigert iron hematoxylin and Biebrich scarlet acid fuchsin solution. After the first stain, the slides were placed in a phosphomolybdic-phosphotungstic acid solution and then stained with aniline blue solution. The stained slides were observed using a light microscope (Leica DM750, Wetzlar, Germany) to detect collagen fibers and nuclei. Immunohistochemistry (IHC) was performed to analyze LAMP-1 and collagen-1 using a UltraVision LP large volume detection system HRP polymer (TL-060-HL, Thermo Scientific) according to the manufacturer’s protocol. LAMP-1/CD107a antibody (1:500, NB120-19294, Novus Biologicals, Centennial, CO, USA) and collagen-1 antibody (1:500, NB600-408, Novus Biologicals) were used as primary antibodies and goat antirabbit IgG secondary antibody (HRP (horseradish peroxidase)) (1:1000, HAF008, R&D Systems, Minneapolis, MN, USA) was used as the secondary antibody.

### 2.12. Enzyme-Linked Immunosorbent Assay

To quantify total level of LAMP-1, HDFs lysate was used. For extraction of total cell lysates, lysis buffer (50 mM Tris-HCl, 150 mM NaCl and 5 mM EDTA, pH 8.0) with a protease and phosphatase inhibitor cocktail (Halt^TM^ Protease inhibitor cocktail, Thermo Fisher Scientific) was used, and the prepared lysate was centrifuged at 13,000× *g* for 20 min at 4 °C. The supernatant obtained from the UVA-irradiated HDFs was used for quantification of secreted cathepsin B, MMP-3 and LAMP-1 using cathepsin B and MMP-3 enzyme-linked immunosorbent assay (ELISA) kits (Abcam, Cambridge, UK) and a LAMP-1 ELISA kit (MyBiosource, San Diego, CA, USA) according to the manufacturers’ instructions. The mice serum was also used to evaluate the production of MMP-2, MMP-3, vascular endothelial growth factor (VEGF) and cathepsin B using mouse MMP2, mouse MMP3 and mouse VEGF ELISA kits (Abcam) and a mouse cathepsin B ELISA kit (Novus Biologicals).

### 2.13. Western Blot Analysis

The dorsal skins were homogenized with RIPA buffer (150 mM sodium chloride, 1% Triton X-100, 1% sodium deoxycholate, 0.1% SDS, 50 mM Tris-HCl, pH 7.5 and 2 mM EDTA) (Biosesang, Seongnam, Korea) and incubated for 30 min at 4 °C. The lysed tissues were centrifuged at 13,500× *g* for 20 min at 4 °C. The separated total protein was collected in a new tube, and the protein concentration was measured using a BCA assay (Thermo Fisher Scientific) according to the manufacturer’s protocol and BSA (Bio-Rad, Hercules, CA, USA) was used as a standard for quantification. Equal protein amounts (40 μg) were separated by 10% SDS-PAGE for LAMP-1 and GAPDH and 8% SDS-PAGE for collagen-1. The proteins on the gel were transferred to PVDF membranes (Millipore, Bedford, MA, USA) using a Trans-Blot semi-dry transfer cell (Bio-Rad). The membranes were placed in 6% nonfat skimmed milk (Neogen, Lansing, MI, USA) in PBS with 0.05% Tween 20 (PBS-T) for 1 h at room temperature and then washed with PBS-T three times for 10 min each followed by treatment with primary antibodies overnight at 4 °C. GAPDH antibody (1:1000, sc-25778, Santa Cruz Biotechnology) was used as an endogenous control. LAMP-1/CD107a antibody (1:500, NB120-19294, Novus Bio) and collagen-1 antibody (1:1000, NB600-408, Novus Bio) were used as primary antibodies. The membranes were washed with PBS-T three times for 10 min and then were reacted with a goat antirabbit IgG secondary antibody (HRP, horseradish peroxidase) (1:2000, HAF008, R&D systems) for 1 h at room temperature. After incubation with the secondary antibody, the membranes were washed with PBS-T, detected using a SuperSignal West Femto Maximum Sensitivity Substrate kit (Thermo Fisher Scientific), and the images were obtained using a FlourChem E (ProteinSimple, San Jose, CA, USA). The protein band was quantified using Image J software (Softomic, Barcelona, Spain).

### 2.14. Statistical Analysis

In vitro data were expressed as the mean ± standard deviation (SD) of three independent experiments. Data of animal tests were expressed as the mean ± standard error (SE) of three independent experiments. The results were evaluated using one-way analysis of the variance (ANOVA), and a posthoc test was performed using the Tukey’s Honest Significant Difference (HSD) method to express significant differences between the groups using the Statistical Package for the Social Science (SPSS) statistical analysis program version 25. A significant difference was indicated when the *p* value was less than 0.05.

## 3. Results

### 3.1. ZLE and Tricin Protect HDFs from UVA-Induced Cell Death and Plasma Membrane Disruption

**Figure 2:**
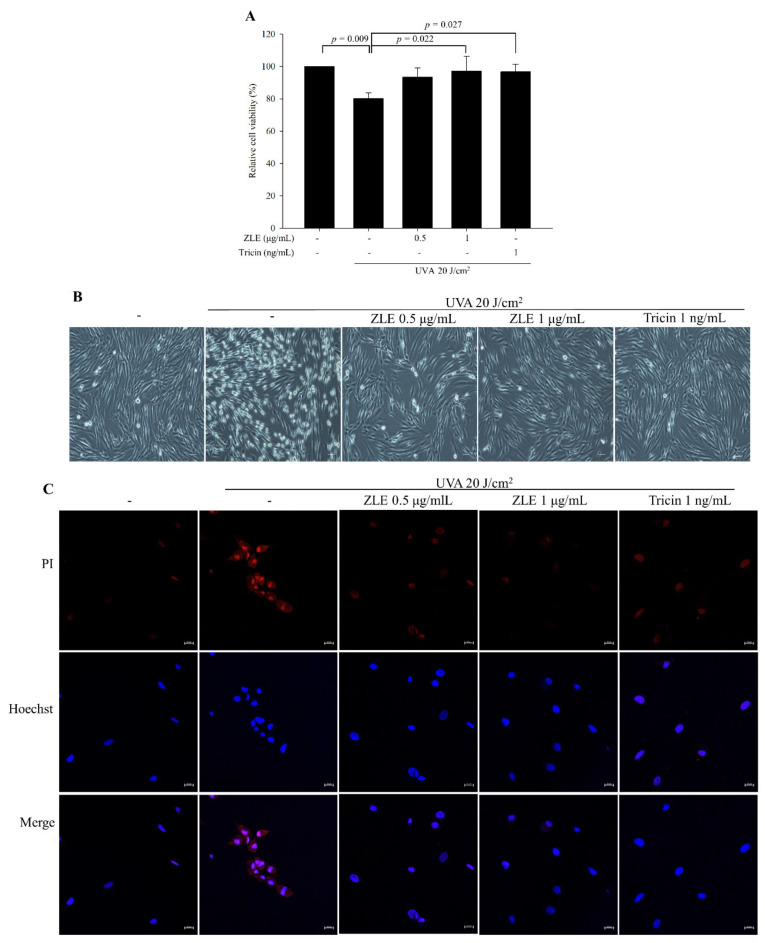
Effects of enzyme-treated *Z. latifolia* extract (ZLE) and tricin on UVA-induced cell viability and plasma membrane disruption in human dermal fibroblasts (HDFs). ZLE and tricin decreased UVA-induced fibroblast cell death and plasma membrane disruption in vitro. A: Effect of ZLE and tricin on UVA-induced cell toxicity in HDFs. HDFs (1 × 10^4^ cells/well) were seeded in 96-well culture plates, and then, the test material was added. After 24 h, the cells were irradiated with UVA (20 J/cm^2^). (**A**) Cell viability was measured by MTT after UVA irradiation. (**B**) UVA-induced plasma membrane disruption in ZLE- and tricin-treated HDFs was observed by microscopy (40× magnification; scale bar, 100 μm). (**C**) UVA-induced plasma membrane disruption in ZLE- and tricin-treated HDFs was measured by propidium iodide staining (20× magnification). The results represent three independent experiments and are expressed as the mean ± SD. The *p* values are determined by ANOVA and Tukey’s HSD test.

To investigate the effect of ZLE and tricin on HDFs subjected to UVA radiation, HDFs were cultured in the presence of ZLE, tricin or vehicle (PBS) for 1 day followed by UVA (20 J/cm^2^) irradiation and culturing for 1 day. Without UVA irradiation, the relative cell viabilities were 100.4% ± 3.6%, 99.7% ± 5.5% and 96.5% ± 6.8% in the HDF cells treated with 0.5 μg/mL ZLE, 1 μg/mL ZLE and 1 ng/mL tricin, respectively, compared with the negative control (100%), which did not show a significant difference. Both the ZLE and tricin treatments followed by UVA irradiation resulted in significantly better cell viability compared with the UVA-irradiated control group (Figure 2A). Disruption of the plasma membrane is a common event after UVA irradiation [22]. As shown in Figure 2B, after UVA irradiation, the plasma membrane was disrupted and propidium iodide (PI) uptake increased, indicating that membrane permeabilization increased (Figure 2C). However, the ZLE and tricin treatments almost completely rescued UVA-induced disruption of the cell membrane and membrane permeabilization. The results suggest that ZLE and tricin protect against UVA by preventing UVA-induced cell membrane rupture.

### 3.2. ZLE and Tricin Suppress UVA-Induced Lysosomal Exocytosis

**Figure 3:**
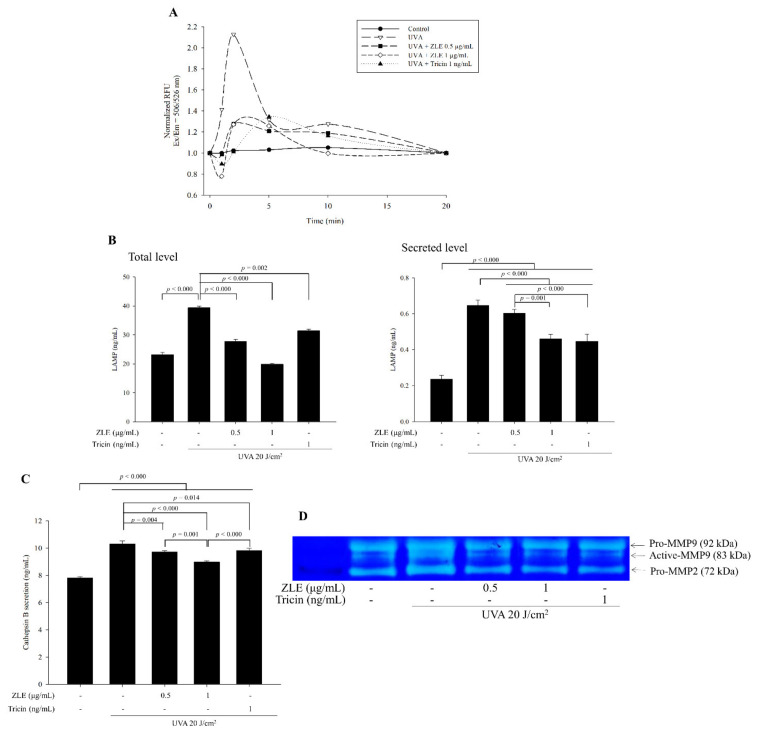
Enzyme-treated *Z. latifolia* extract (ZLE) and tricin suppress calcium influx and lysosomal exocytosis caused by UVA irradiation in vitro. (**A**) Human dermal fibroblasts (HDFs) were irradiated with UVA followed by ZLE or tricin treatment, and then, calcium influx was measured by Fluo-4 staining. (**B**) Secretion of LAMP was measured by ELISA. (**C**) Secretion of cathepsin B was measured by ELISA. (**D**) Gelatin zymography for UVA-induced pro- and active-MMPs in HDFs. The results represent three independent experiments and are expressed as the mean ± SD. The *p* values are determined by ANOVA and Tukey’ HSD test.

Lysosomal associated membrane proteins (LAMPs) have roles in intracellular transport of hydrolases and the regulation of membrane fusion events. It was previously shown that UVA irradiation caused localization of LAMPs into the plasma membrane and induced lysosomal exocytosis followed by calcium ion influx from the extracellular matrix through a damaged cell membrane [22]. Calcium ion influx through the damaged cell membrane increased after UVA irradiation, while treatments with ZLE and tricin induced decreased calcium ion influx after UVA irradiation (Figure 3A). Following the increase in calcium ion influx, the UVA irradiation also caused increases in total and secreted LAMPs. Treatment of the HDFs with ZLE and tricin significantly lowered the total and secreted LAMPs compared with the UVA-irradiated control group (Figure 3B).

Lysosomal exocytosis induces protease secretion from the lysosome, which causes ECM destruction resulting in the secretion of many growth factors from the ECM, such as VEGF and FGF, among others [23]. Expression of cathepsin B was not changed by UVA irradiation or UVA irradiation with ZLE and tricin, while UVA irradiation in HDF cells increased cathepsin B secretion compared with the negative control, and its secretion was significantly reduced by both the ZLE and tricin treatments (Figure 3C). These results suggest that the ZLE and tricin treatments in UVA-irradiated cells did not change the expression of cathepsin B but changed its secretion. Cathepsin B is known to activate pro-MMPs such as MMP-2 and 9, which in turn, target the ECM resulting in ECM degradation and wrinkle formation [24]. From gelatin zymography, it was evident that UVA irradiation resulted in increased MMP-2 and 9 expression and in particular, increased MMP-9 activation (Figure 3D). Treatment with ZLE and tricin significantly decreased MMP-2 and 9 expression and activation of MMP-9 induced by UVA irradiation. The results suggest that ZLE and tricin lowered UVA-induced lysosomal exocytosis, thus reducing the release and activation of these proteases.

### 3.3. ZLE and Tricin Alleviate UVA-Induced Oxidative Stress

**Figure 4:**
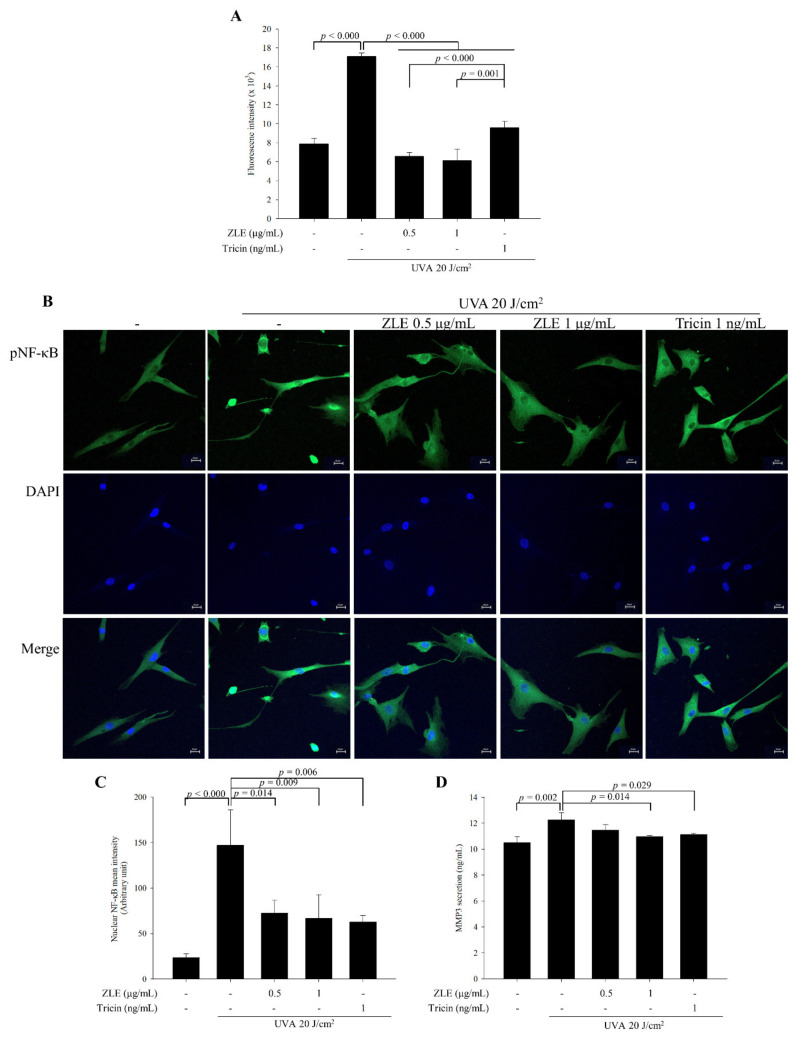
Enzyme-treated *Z. latifolia* extract (ZLE) and tricin alleviate oxidative stress and the effects on the downstream cascade caused by UVA irradiation in vitro. (**A**) Effect of ZLE and tricin on UVA-induced reactive oxygen species (ROS) generation in human dermal fibroblasts (HDFs). (**B**,**C**) Effect of ZLE and tricin on UVA-induced activation of NF-κB in HDFs. Representative images at 20× magnification. (**D**) Secretion level of MMP3 was quantified by ELISA. The results represent three independent experiments and are expressed as the mean ± SD. The *p* values are determined by ANOVA and Tukey’s HSD test.

UVA-induced ROS and related oxidative stress mediate cellular photosensitization and induce wrinkle formation. ROS are the main activators of NF-κB through phosphorylation of its subunits resulting in the promotion of its DNA binding affinity and upregulation of the expression of MMPs [6,7]. The ROS in the UVA-irradiated HDFs increased by 116.7% compared with the UVA-untreated group. By comparison, in the UVA-irradiated HDFs treated with 0.5 μg/mL ZLE, 1 μg/mL ZLE and 1 μg/mL tricin, the ROS decreased by 61.5%, 64.2% and 44.0%, respectively, compared with the UVA-irradiated control group (Figure 4A). NF-κB activation was induced by UVA irradiation compared with the UVA-untreated HDFs and treatment of ZLE or tricin inhibited NF-κB activation (Figure 4B,C). Following the NF-κB activation in the UVA-irradiated group, the MMP-3 secretion level was upregulated, while treatments with ZLE and tricin induced downregulation of the MMP-3 secretion level (Figure 4D). The results suggest that ZLE and tricin inhibit oxidation and its cascade effects such as NF-κB activation and MMP-3 expression.

### 3.4. Body Weight and Serum Analysis in UVA-Irradiated SKH-1 Hairless Mice Treated with ZLE

**Figure 5:**
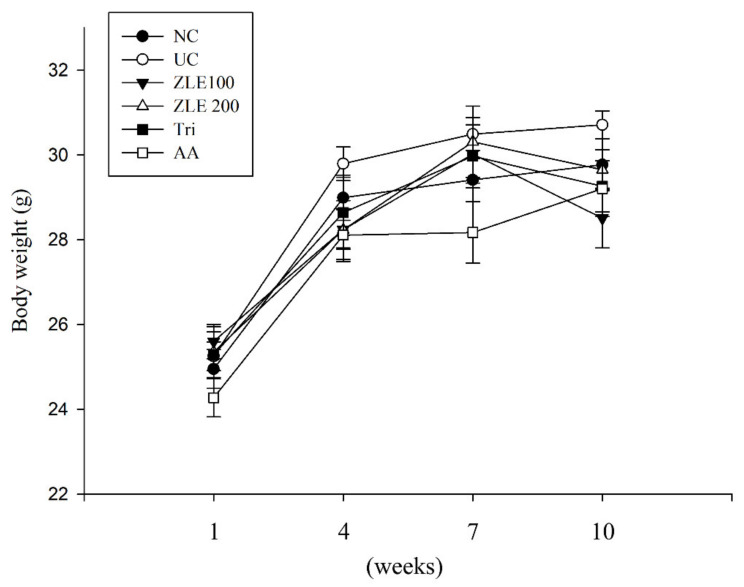
Effects of enzyme-treated *Z. latifolia* extract (ZLE) and tricin on body weight in UVA-irradiated mice. The body weight was measured every 3 weeks. Data are expressed as the mean ± SE.

Based on the results of the in vitro experiments, the antiwrinkle effects of ZLE and tricin were assessed in UVA-irradiated SKH-1 hairless mice. To investigate the effect of administration of ZLE, tricin and ascorbic acid for 10 weeks, body weight and serum GOT and GPT were measured. Body weight was measured at 1, 4, 7 and 10 weeks. There was no significant difference in body weight among the groups during the experiment (Figure 5). To examine the hepatotoxicity of ZLE, tricin and ascorbic acid, serum GOT and GPT were measured after 10-week oral administration. The results show that there were no significant differences in GOT and GPT in the various treatment groups compared with the normal control (Table 1). The results suggest that oral administration of ZLE, ascorbic acid and tricin did not significantly affect body weight and hepatotoxicity in UVA-irradiated mice.

### 3.5. ZLE and Tricin Protect the Skin Surface in UVA-Irradiated SKH-1 Hairless Mice

**Figure 6:**
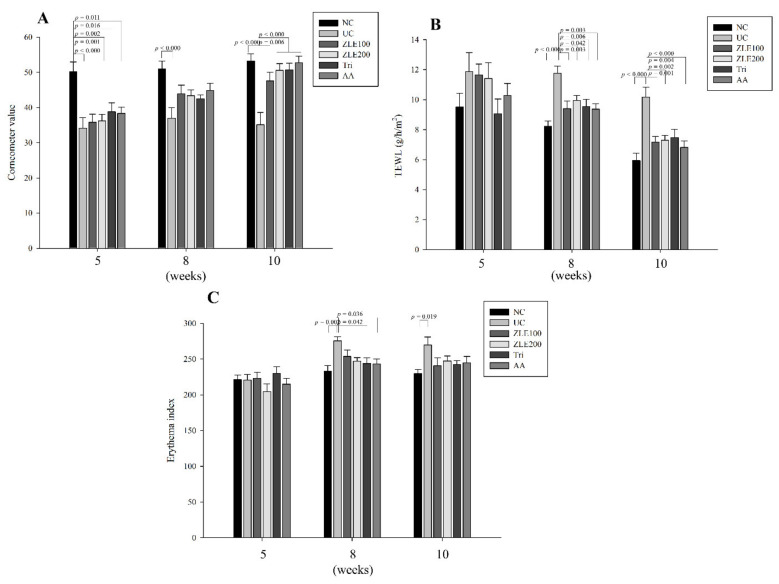
Effects of enzyme-treated *Z. latifolia* extract (ZLE) and tricin on dermal moisture content, transepidermal water loss (TEWL) and the dermal erythema index of UVA-irradiated mice dorsal skin. (**A**) Dermal moisture content, (**B**) TEWL and (**C**) dermal erythema index. Data are expressed as the mean ± SE. The *p* values are determined by ANOVA and Tukey’s HSD test.

To examine the effects of ZLE and tricin on skin damaged by UVA irradiation, dermal moisture content, TEWL and the dermal erythema index of UVA-irradiated dorsal skin in SKH-1 hairless mice were measured at 5, 8 and 10 weeks after treatment with ZLE and tricin. The dermal moisture content of the NC, UC, ZLE100, ZLE200, Tri and AA groups was 50.2 ± 2.8, 34.1 ± 3.0, 35.8 ± 2.3, 36.3 ± 1.5, 38.8 ± 2.5 and 38.3 ± 1.8 AU (arbitrary unit), respectively, at 5 weeks after treatment; 51 ± 2.3, 36.9 ± 3.1, 44 ± 2.4, 43.4 ± 2.1, 42.4 ± 1.1 and 44.8 ± 1.6 AU, respectively, at 7 weeks after treatment; and 53.3 ± 2.0, 35.1 ± 3.5, 47.6 ± 2.5, 50.6 ± 1.9, 50.7 ± 1.9 and 52.7 ± 1.9 AU, respectively, after 10 weeks of treatment. The results show that the dermal moisture content of UC significantly decreased by 47%, 38% and 51.5% at 5, 7 and 10 weeks after treatment, respectively, compared with the NC. The dermal moisture content in the ZLE100, ZLE200, Tri and AA groups also significantly increased by 35.4%, 44%, 44.4% and 44.9%, respectively, at 10 weeks after treatment compared with the UC (Figure 6A).

The TEWLs of the NC, UC, ZLE100, ZLE200, Tri and AA groups were 9.5 ± 0.9, 11.9 ± 1.3, 11.7 ± 0.7, 11.4 ± 1.1, 9.1 ± 1 and 10.3 ± 0.8 g/h/m^2^, respectively, at 5 weeks after treatment; 8.2 ± 0.4, 11.8 ± 0.5, 9.4 ± 0.5, 10 ± 0.3, 9.5 ± 0.5 and 9.4 ± 0.4 g/h/m^2^, respectively, at 8 weeks after treatment; and 5.9 ± 0.5, 10.2 ± 0.7, 7.2 ± 0.4, 7.3 ± 0.3, 7.5 ± 0.5 and 6.8 ± 0.4 g/h/m^2^, respectively, at 10 weeks after treatment. The results indicate that the TEWL of the UC significantly increased by 30.2% and 41.5% at 8 and 10 weeks, respectively, compared with the NC. The results of TWELs in the ZLE100, ZLE200, Tri and AA groups significantly decreased by 20.1%, 15.5%, 18.9% and 20.4%, respectively, at 8 weeks and by 29.3%, 28.1%, 26.4% and 32.9%, respectively, at 10 weeks compared with the UC (Figure 6B).

The erythema indices of the NC, UC, ZLE100, ZLE200, Tri and AA groups were 221 ± 6.9, 220.4 ± 8.3, 223.1 ± 8.2, 204.5 ± 10.6, 229.9 ± 9.6 and 214.8 ± 8, respectively, at 5 weeks after treatment; 232.7 ± 8.3, 275.4 ± 5.9, 253.6 ± 9.2, 246.8 ± 5.3, 243.6 ± 8.3 and 243 ± 6.9, respectively, at 8 weeks after treatment; 229.6 ± 5.7, 269.8 ± 11, 240.5 ± 10.9, 247.3 ± 7, 242.3 ± 5.7 and 244.5 ± 9.2, respectively, at 10 weeks treatment. The results of the erythema indices indicated that the erythema of the UC significantly increased by 15.5% and 14.9% at 8 and 10 weeks, respectively, compared with the NC. The results of erythema indices in the ZLE200 and Tri groups significantly decreased by 10.4% and 11.5%, respectively, at 8 weeks compared with the UC (Figure 6C). UVA irradiation negatively affects the skin surface by reducing moisture content and increasing TEWL and erythema. The results suggest that surface damage of the mice dorsal skin by UVA irradiation was effectively protected by oral administration of ZLE, AA and tricin.

### 3.6. ZLE and Tricin Suppress Wrinkle Formation in UVA-Irradiated SKH-1 Hairless Mice

**Figure 7:**
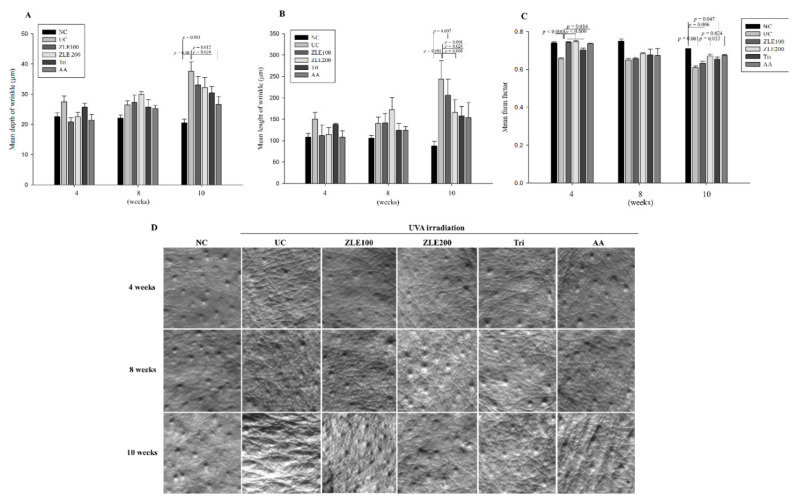
Effects of enzyme-treated *Z. latifolia* extract (ZLE) and tricin on the prevention of wrinkle formation in UVA-irradiated mice. The wrinkles on replica skin were measured by a Visioline VL650. (**A**) Mean depth of wrinkles. (**B**) Mean length of wrinkles. (**C**) Mean form factor. (**D**) Representative images of replica skin. Data are expressed as the mean ± SE. The *p* values are determined by ANOVA and Tukey’s HSD test.

To evaluate the effects of ZLE and tricin on the prevention of wrinkle formation, the mean depth of wrinkles, mean length of wrinkles and mean form factor were measured in the replica of the UVA-irradiated SKH-1 hairless mice dorsal skin by using a Visiloine VL650. The mean depth of wrinkles in the NC, UC, ZLE100, ZLE200, Tri and AA groups was 22.5 ± 1.4, 27.4 ± 1.9, 20.8 ± 1.4, 25.7 ± 1.3 and 21.5 ± 1.8 μm, respectively, at 4 weeks after treatment; 22.1 ± 0.9, 26.4 ± 1.4, 27.3 ± 2.3, 30 ± 0.8, 25.8 ± 2.4 and 25.3 ± 1 μm, respectively, at 8 weeks after treatment; and 20.5 ± 1.3, 37.6 ± 3, 33.1 ± 2.7, 32.2 ± 3.4, 30.4 ± 2.1 and 26.6 ± 2.5 μm, respectively, at 10 weeks after treatment. The results indicate that the mean depth of wrinkles in the UC significantly increased by 45.6% at 10 weeks compared with the NC; however, the mean depth of wrinkles of the Tri and AA groups significantly decreased by 19.2% and 29.2%, respectively, at 10 weeks compared with the UC (Figure 7A).

The mean length of wrinkles in the NC, UC, ZLE100, ZLE200, Tri and AA groups was 108 ± 8.6, 150 ± 16.4, 112 ± 24, 114 ± 16.3, 138 ± 2 and 108 ± 14.6 μm, respectively, at 4 weeks of treatment; 106 ± 6, 140 ± 14.8, 142 ± 21.3, 172 ± 28.2, 124 ± 16.3 and 134 ± 9.3 μm, respectively, at 8 weeks of treatment; and 88 ± 10.2, 244 ± 42.8, 206 ± 37.7, 166 ± 28.9, 158 ± 21.8 and 154 ± 34.7 μm, respectively, at 10 weeks of treatment. The mean length of wrinkles in the UC significantly increased by 63.9% at 10 weeks compared with the NC; however, the mean length of wrinkles of the ZLE200, Tri and AA groups significantly decreased by 32%, 35.2% and 36.9%, respectively, at 10 weeks compared with the UC (Figure 7B).

The mean form factor is the degree of overall wrinkles in the replica, and the closer it is to 1, the less wrinkles are formed. The results of the mean form factor analysis in NC, UC, ZLE100, ZLE200, Tri and AA groups were 0.74 ± 0.008, 0.66 ± 0.005, 0.74 ± 0.003, 0.75 ± 0.003, 0.7 ± 0.005 and 0.74 ± 0.003, respectively, at 4 weeks of treatment; 0.75 ± 0.012, 0.65 ± 0.008, 0.66 ± 0.005, 0.68 ± 0.005, 0.68 ± 0.003 and 0.67 ± 0.036, respectively, at 8 weeks of treatment; and 0.71, 0.61 ± 0.008, 0.63 ± 0.011, 0.67 ± 0.011, 0.65 ± 0.012 and 0.67 ± 0.007, respectively, at 10 weeks of treatment. The mean form factor in the UC significantly decreased by 12.7% and 16.4% at 4 and 10 weeks, respectively, compared with the NC; however, the mean form factor of the ZLE100, ZLE200, Tri and AA groups significantly increased by 13.2%, 13.7%, 7.1% and 12.2%, respectively, at 4 weeks, and the mean form factor of the ZLE200 and AA groups significantly increased by 9.8% and 10.4%, respectively, at 10 weeks compared with the UC (Figure 7C). Representative images for each group are shown in Figure 7D. These results suggest that oral administration of ZLE, tricin and ascorbic acid all effectively prevent UVA-induced wrinkle formation.

### 3.7. Effects of ZLE and Tricin on Secretion of MMP-2, MMP-3, VEGF and Cathepsin B in the Serum of UVA-Irradiated SKH-1 Hairless Mice

**Figure 8:**
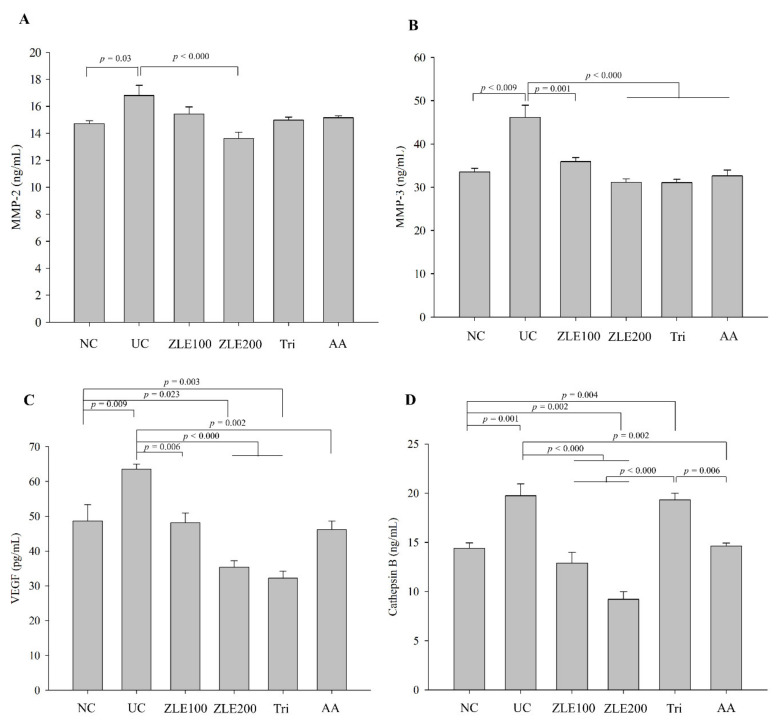
Effects of enzyme-treated *Z. latifolia* extract (ZLE) and tricin on the secretion of MMP-2, MMP-3, VEGF and cathepsin B in serum by UVA irradiation. (**A**) serum MMP-2; (**B**) serum MMP-3; (**C**) serum VEGF; (**D**) serum cathepsin B. The serum levels of the proteins were measured by ELISA. Data are expressed as the mean ± SE. The *p* values are determined by ANOVA and Tukey’s HSD test.

MMP-2 and MMP-3, known as type Ⅳ collagenase and stromelysin-1, respectively, cause degradation of extracellular matrix proteins as a result of photoaging by UV-irradiation [25,26]. The enzyme activities of MMPs are upregulated through induction of photooxidative stress by UVA irradiation [27]. To determine the effects of the oral administration of ZLE, ascorbic acid and tricin on secretion of MMP-2, MMP-3, VEGF and cathepsin B in UVA-irradiated mice, MMP-2, MMP-3, VEGF and cathepsin B were measured in the serum of each group by using ELISA. The serum MMP-2 levels in the NC, UC, ZLE100, ZLE200, Tri and AA groups were 14.7 ± 0.2, 16.8 ± 0.7, 15.4 ± 0.5, 13.6 ± 0.5, 15 ± 0.2 and 15.2 ± 0.1 ng/mL, respectively, and the serum MMP-3 levels in the NC, UC, ZLE100, ZLE200, Tri and AA groups were 33.5 ± 0.8, 46.1 ± 2.8, 35.9 ± 0.9, 31.1 ± 0.8, 31.1 ± 0.8 and 32.6 ± 1.3 ng/mL, respectively (Figure 8A,B). The results showed that the serum MMP-2 and MMP-3 levels of the UC significantly increased by 14.2% and 37.5%, respectively, compared with the NC. The serum MMP-2 level significantly decreased in the ZLE200 group compared with the UC, and the serum MMP-3 level significantly decreased in the ZLE100, ZLE200, AA and Tri groups compared with the UC.

VEGF is distinctly upregulated by UV irradiation in keratinocytic cells [28]. The serum VEGF levels in the NC, UC, ZLE100, ZLE200, Tri and AA groups were 48.7 ± 4.6, 63.6 ± 1.4, 48.1 ± 2.8, 35.4 ± 1.9, 32.2 ± 2 and 46.2 ± 2.4 pg/mL, respectively (Figure 8C). The serum VEGF level of the UC significantly increased by 23.4% compared with the NC. The serum VEGF levels significantly decreased in the ZLE100, ZLE200, AA and Tri groups compared with the UC. Cathepsin B processes pro-MMPs to active MMPs that, in turn, degrade the extracellular matrix [24]. Cathepsin B also functions as a cysteine protease and a proapoptotic mediator when it is released from lysosomes by UVA irradiation [29]. The serum cathepsin B levels in the NC, UC, ZLE100, ZLE200, Tri and AA groups were 14.4 ± 0.6, 19.8 ± 1.2, 12.9 ± 1.1, 9.2 ± 0.8, 19.3 ± 0.7 and 14.6 ± 0.3 ng/mL, respectively (Figure 8D). The serum cathepsin B level of the UC significantly increased by 27.1% compared with the NC group, while the serum cathepsin B levels significantly decreased in the ZLE100, ZLE200 and AA groups compared with the UC.

### 3.8. ZLE and Tricin Protect Skin Damage in UVA-Irradiated SKH-1 Hairless Mice

**Figure 9:**
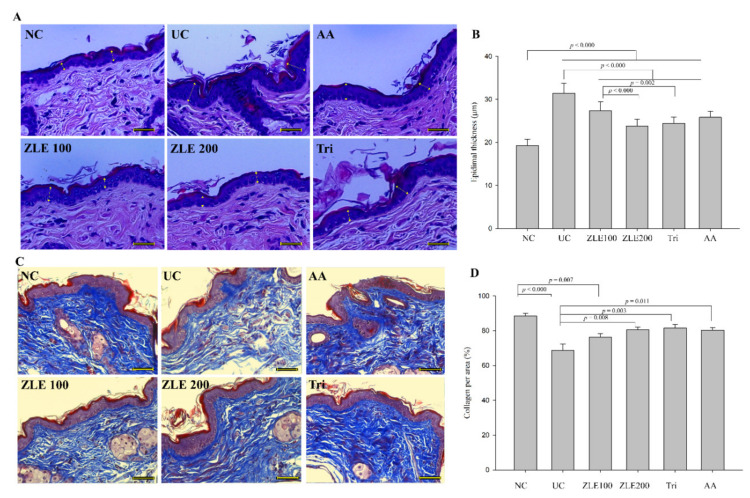
Effects of enzyme-treated *Z. latifolia* extract (ZLE) and tricin on the reduction in epidermal thickness and accumulation of collagen fiber in UVA-irradiated mice. The skin sections were stained using H&E staining or Masson’s trichrome staining. (**A**): Photographs of representative H&E stained skin sections. (**B**): Thickness of mice skin epidermis. (**C**): Representative Masson’s trichrome stained skin sections. (**D**): Quantification of collagen-positive pixels (blue color). The scale bar, located on the lower right of each image, is 0.1 mm and the images were obtained at 200× magnification. Data are expressed as the mean ± SE. The *p* values are determined by ANOVA and Tukey’s HSD test.

UV irradiation causes damage to signal pathways, which induces increased keratinocyte cell division resulting in the increase in epidermal thickness [1]. Both UVA and UVB cause alteration of the epidermal thickness [30]. To investigate the effect of the oral administration of ZLE and tricin on epidermal thickness, dermal skins were stained with H&E, and the keratinocyte thickness was measured. The epidermal thickness in the NC, UC, ZLE100, ZLE200, Tri and AA groups was 19.3 ± 1.4, 31.4 ± 2.3, 27.4 ± 2, 23.8 ± 1.6, 24.4 ± 1.5 and 25.8 ± 1.4 μm, respectively (Figure 9A,B). The epidermal thickness of the UC significantly increased by 38.7% compared with the NC. However, the epidermal thickness of the ZLE100, ZLE200, AA and Tri groups significantly decreased compared with the UC.

To examine the effect of the oral administration of ZLE and tricin on the morphology of collagen fiber, dorsal skin was stained by Masson’s trichrome staining, which stains keratin red and collagen fiber blue. The % area of collagen fiber in the dorsal skin was analyzed by measuring the area that was stained blue and dividing it by the total tissue area as per the following formula: % area of collagen fiber = (blue-stained area/total tissue area) × 100. The % areas of collagen fiber in the NC, UC, ZLE100, ZLE200, Tri and AA groups were 88.4% ± 1.6%, 68.6% ± 3.7%, 76.3% ± 2%, 80.6% ± 1.5%, 81.6% ± 1.9% and 80.3% ± 1.6%, respectively (Figure 9C,D). The % area of collagen fiber in the UC significantly decreased compared with the NC. By comparison, the % area of collagen fiber significantly increased in the ZLE200, Tri and AA groups. The results suggest that the administration of ZLE, tricin and AA all improve the damaged epidermal tissue and inhibit the destruction of dermal collagen fibers by UVA irradiation, possibly by preventing the dermal matrix from breakdown by MMPs, VEGF and cathepsin B.

### 3.9. ZLE and Tricin Alter Expression of LAMP-1 and Collagen-1 in UVA-Irradiated SKH-1 Hairless Mice

**Figure 10:**
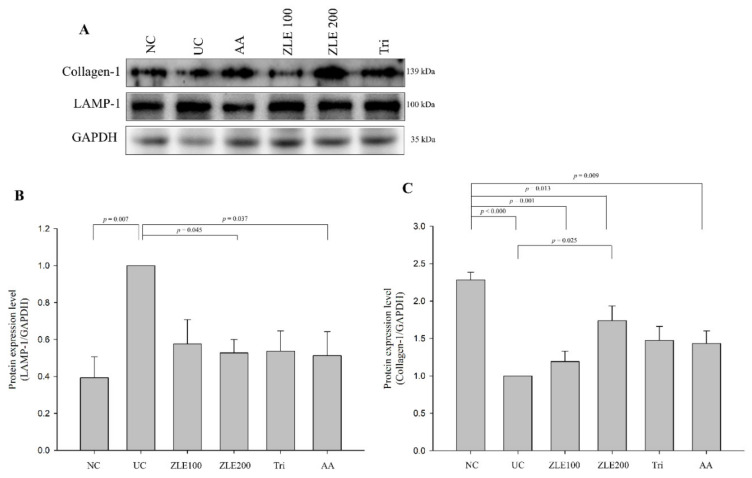
Effects of enzyme-treated *Z. latifolia* extract (ZLE) and tricin on the expression of LAMP-1 and collagen-1 in the UVA-irradiated skin tissue. The expression levels of LAMP-1 and collagen-1 were measured by Western blotting. GAPDH was used as an endogenous control. (**A**): Representative images of Western blot analysis. (**B**): Quantification of LAMP-1. (**C**): Quantification of collagen-1. Data are expressed as the mean ± SE. The *p* values are determined by ANOVA and Tukey’s HSD test.

To examine the effects of ZLE and tricin on the protein expression of LAMP-1 and collagen-1, the proteins were extracted from the dorsal skin of mice and measured by Western blotting. The fold changes of LAMP-1 and collagen-1 expression in the UC were 2.28 ± 0.11 and 0.38 ± 0.11, respectively, compared with the NC (Figure 10A). The fold changes of LAMP-1 expression levels in the ZLE100, ZLE200, Tri and AA groups were 0.58 ± 0.13, 0.53 ± 0.07, 0.54 ± 0.11 and 0.51 ± 0.13, respectively, compared with the UC (Figure 10B). The expression levels of LAMP-1 in the ZLE200 and AA groups significantly decreased compared with the UC. The fold changes of collagen-1 expression levels in the ZLE100, ZLE200, Tri and AA groups were 1.19 ± 0.14, 1.74 ± 0.2, 1.47 ± 0.19 and 1.43 ± 0.17, respectively, compared with the UC (Figure 10C). The expression level of collagen-1 in the ZLE200 significantly increased compared with the UC. The results showed that the protein expression levels of LAMP-1 and collagen-1 were altered in the dorsal skin tissue by UVA irradiation. The results suggest that the administration of ZLE, tricin and AA all inhibit lysosomal exocytosis induced by UVA irradiation.

### 3.10. ZLE and Tricin Alter the Localization of LAMP-1 and Collagen-1 in UVA-Irradiated SKH-1 Hairless Mice

**Figure 11:**
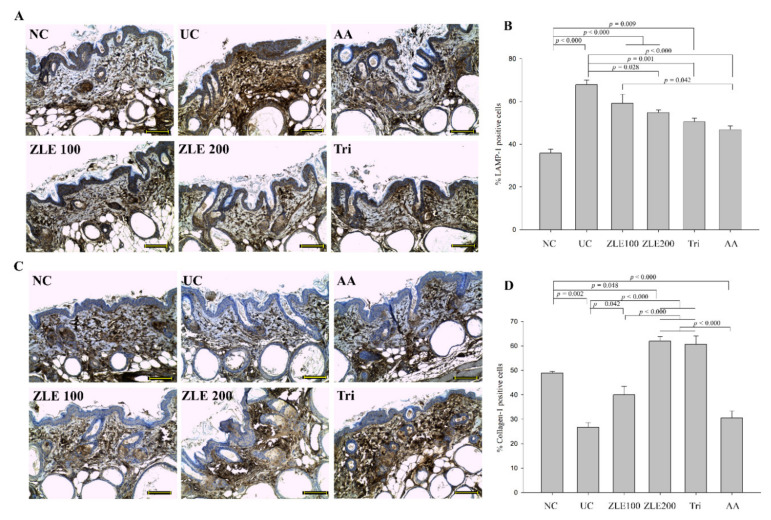
Effects of enzyme-treated *Z. latifolia* extract (ZLE) and tricin on the localization of LAMP-1 and collagen-1 in the UVA-irradiated dermis. (**A**): Representative IHC images of LAMP-1 in the skin sections (brown color). (**B**): Quantification of LAMP-1 positive cells (%). (**C**): Representative IHC images of collagen-1 in the skin sections (brown color). (**D**): Quantification of collagen-1 positive cells (%). The scale bar, located in the lower right of each image, is 0.1 mm, and the sections were obtained at 200× magnification. Data are expressed as the mean ± SE. The *p* values are determined by ANOVA and Tukey’s HSD test.

Lysosomal exocytosis contributes to the formation of wrinkles and skin damage by degradation of the ECM. Accordingly, the measurement of alterations in the localization of LAMP-1 and collagen-1 are important to evaluate the antiwrinkle effects of ZLE and tricin. IHC was performed to evaluate the localization and accumulation of LAMP-1 and collagen-1 in the mice skin tissue. LAMP-1 in the NC was mainly localized below the dermis in the area known as the subcutaneous tissue (also called the hypodermis), whereas the localization of LAMP-1 in the UA was generally increased in the dermis (Figure 11A). The LAMP-1 positive cells (%) in the NC, UC, ZLE100, ZLE200, Tri and AA groups were 35.8% ± 1.9%, 67.8% ± 2.3%, 59.1% ± 4.2%, 54.9% ± 1.1%, 50.5% ± 1.7% and 46.8% ± 1.6%, respectively (Figure 11B). The LAMP-1 positive cells located in the dermis in the UC significantly increased compared with the NC group. By comparison, the LAMP-1 positive cells significantly decreased in the ZLE200, AA and Tri groups.

The collagen-1 of the UC group was reduced in the dermis layer compared with the NC (Figure 11C). The collagen-1 positive cells (%) in the NC, UC, ZLE100, ZLE200, Tri and AA groups were 48.9% ± 0.7%, 26.7% ± 1.9%, 40% ± 3.4%, 61.9% ± 1.8%, 60.6% ± 3.4% and 30.5% ± 2.9%, respectively (Figure 11D). The results suggest that the collagen-1 was damaged by UVA irradiation; however, oral administration of ZLE (100 and 200 mg/kg/day) and tricin protected collagen-1 in the skin.

LAMP-1 composes 50% of the lysosomal membrane proteins [31]. As shown by the results of the in vitro experiment, the total and secreted levels of LAMP-1 increased by UVA irradiation. These results suggest that lysosome exocytosis occurred followed by the alteration of localization of LAMP-1 by UVA irradiation. Additionally, a reduction in collagen-1 was clearly observed in the dermal tissue following UVA irradiation. The results suggest that the changes in the skin induced by UVA irradiation were restored by administration of ZLE, tricin and AA.

## 4. Discussion

Skin aging is the sum of three distinct sets of drivers: intrinsic aging; a group of metabolic, nutritional and lifestyle factors such as smoking, which are modifiable via dietary and lifestyle changes; and photoaging. Intrinsic aging results in mainly functional alterations and occurs naturally by genetically induced physiological changes. Metabolic stress is commonly induced by diabetes, where ECM proteins in skin and other tissues are subject to glycative and oxidative damage [32]. Nutritional factors include intakes of tocopherols, tocotrienols, carotenoids, xanthophyls and polyphenols which variously enter the skin and modify UV absorption, oxidation, inflammation and DNA repair [33,34,35]. Generally, photoaging is caused by exposure to UVA and UVB irradiation. Skin aging processes result in different appearances in terms of wrinkle formation [36]. In contrast with intrinsic skin aging, photoaging tends to induce deep wrinkles, roughness on the skin and extension of capillaries [37]; however, the mechanism of photoaging has not been fully defined. In particular, limited information is available on wrinkle formation through ROS generation and lysosomal exocytosis by UVA irradiation compared with wrinkle formation by UVB irradiation. In addition to the known antioxidant effects of ZLE [38], it also showed an antiwrinkle effect on UVB-irradiated HDFs and SKH-1 hairless mice by blocking the MAPK signaling pathway resulting in the inhibition of NF-κB and activator protein-1 (AP-1) activities [18,19]. Both ZLE and tricin treatments showed skin protective effects and helped reduce wrinkle formation in UVA-irradiated human dermal fibroblasts and hairless mice in this study.

UVB is absorbed by the epidermis skin layer and causes burning, while UVA is absorbed by the dermis skin layer and causes aging and wrinkling via diverse UV-induced damage such as degradation of collagen, pigmentation and DNA mutations [39]. The main mechanisms of UVA-induced wrinkle formation are ROS generation and lysosomal exocytosis, which are reduced by ZLE and tricin. When the plasma membrane of a skin fibroblast is damaged, lysosomes combine with the torn plasma membrane and use Ca^2+^ influx from the extracellular components to restore the membrane. Extracellular Ca^2+^ is needed to expose LAMP-1 on the cell surface. LAMP-1 does not normally localize in the plasma membrane, but does localize in the plasma membrane under conditions when the cell is wounded. This localization causes lysosomal exocytosis and also releases cysteine proteases such as cathepsins B, L and K that degrade the ECM and are key players in the promotion of melanoma proliferation [40]. UVA irradiation can induce the activation of endogenous photosensitizers that produce ROS, which causes oxidative stress through DNA damage. The induction of ROS results in serious damage to the proteome [3,29,41]. In this study, UVA irradiation caused rupture of the plasma membrane followed by ROS generation and secretion of LAMP-1 with an influx of Ca^2+^ in HDFs. In the in vivo experiment in mice, the location and amount of LAMP-1 was significantly different between the negative control and the UVA-irradiated groups in the skin tissue. LAMP-1 was found throughout the epidermis in the UVA-irradiated skin, and the secretion and protein expression of LAMP-1 increased in the UVA-irradiated HDFs and skin tissue. ZLE and tricin decreased the secretion of LAMP-1. Lysosomal exocytosis, which is detected by the localization of LAMP-1 into the extracellular matrix, causes secretion of lysosomal proteases such as cathepsin B from the cytosolic compartment to the extracellular compartment [42]. The total LAMP-1 levels of all groups except the UVA-irradiated control group were similar, which indicates that ZLE and tricin inhibited the expression of LAMP-1 in UVA-irradiated HDFs. In this study, secretion of LAMP-1 increased in HDFs after UVA irradiation, which might be because of the rupture of the plasma membrane by UVA irradiation. However, secretion of LAMP-1 decreased by treatment with ZLE and tricin in UVA-irradiated HDFs. Treatments with ZLE and tricin resulted in similar outcomes in UVA-irradiated hairless mouse skin. Therefore, ZLE and tricin prevent skin aging by preventing lysosomal exocytosis. Although both ZLE and tricin showed the protective effects on UVA-irradiated skin tissue, in general, ZLE showed more protective effects than tricin at the concentrations used in this study.

The main mechanism responsible for photoaging caused by stimulation of ROS is the depletion and damage of the nonenzymatic and enzymatic antioxidant defense systems of the skin [43,44,45], while little information on other mechanisms of photoaging related to the action of lysosomes is available. Increased ROS by UVA irradiation causes DNA damage followed by upregulation of expression of genes related to the MAPK signaling pathway such as c-Jun, AP-1 and NF-κB [46]. In this study, we found that UVA irradiation causes lysosomal exocytosis as well as a high amount of ROS generation, which were improved by treatment with ZLE and tricin. Protein expression of activated NF-κB and the secretion levels of MMP-2, -3 and -9 in HDFs significantly increased by irradiation with UVA, which decreased cell viability and increased plasma membrane disruption. Treatments with ZLE and tricin decreased protein levels of active MMP-2 and -9. An increase in secreted cathepsin B has been shown to increase the level of active MMP-2 and -9 through its proteolytic activity [47]. In our in vivo experiment, the secretion levels of cathepsin B, MMPs and VEGF significantly increased after UVA irradiation. Therefore, UVA irradiation induces photoaging in HDFs and hairless mice. The production of diverse factors such as MMPs, cathepsins and growth factors that breakdown the ECM is accelerated because of the stimulation of the MAPK signaling pathway. At the same time, exocytosis of destabilized lysosomes is also accelerated, and consequently, ECM degradation occurs. The degradation of destabilized lysosomes is called lysosomal shedding or ectodomain shedding, which functions in proteolytic cleavage of plasma membrane proteins and causes protein turnover. Although the action of lysosomes is essential to regulate the development of the cell, the immune response and homeostasis, excessive lysosomal shedding causes degradation of the ECM [48].

Wrinkle formation was intensified, and collagen-1 decreased in the skin tissue of UVA-irradiated hairless mice. In addition to the positive effects of ZLE observed for the UVB-irradiated condition, ZLE and tricin effectively provided protection from skin damage caused by UVA. More importantly, ZLE and tricin played a significant role in the inhibition of lysosomal shedding in UVA-irradiated skin resulting in the prevention of wrinkle formation. Therefore, ZLE and tricin inhibit ROS generation and secretion/localization of LAMP-1, which results in the inhibition of lysosomal exocytosis and prevention of wrinkle formation. 

## 5. Conclusions

UVA is one of the main causes of wrinkle formation by inducing ROS and lysosome destabilization. Destabilized lysosomes migrate to the ECM and increase cathepsin B that, in turn, stimulates conversion of pro-MMPs to MMPs, which alters the ECM environment and ultimately results in the destruction of the ECM and breakdown of the dermal matrix. Destruction and inadequate turnover of the ECM facilitates wrinkle formation of the skin. In this study, we found that ZLE and tricin provide an antiwrinkle effect. ZLE and tricin suppress HDF apoptosis and ROS generation by UVA irradiation. Moreover, the secretion of cathepsin B and the protein expression of downstream factors such as MMPs and NF-κB decrease in UVA-irradiated HDFs treated with ZLE and tricin. Additionally, oral administration of ZLE and tricin to UVA-irradiated hairless mice effectively protects skin from damage by preserving the moisture of the skin surface. Collagen fiber and the epidermal condition are also conserved in UVA-irradiated hairless mice by oral administration of ZLE and tricin. Additionally, the secretion of MMPs, VEGF and cathepsin B and the translocation of LAMP-1 and collagen-1 decrease in UVA-irradiated hairless mice orally administered ZLE and tricin. In conclusion, ZLE and tricin inhibit skin damage and prevent wrinkle formation through the inhibition of ROS generation and lysosomal exocytosis.

## Figures and Tables

**Table 1:** Effect of enzyme-treated *Z. latifolia* extract (ZLE) and tricin on hepatotoxicity biomarkers in UVA-irradiated mice.

Biomarker	Normal Control	UVA Control	ZLE 100	ZLE 200	Tri	AA
	(U/L)					
GOT	124.8 ± 17.7	147.9 ± 17.2	125.6 ± 17.8	132.4 ± 11.2	126.9 ± 16.4	149.7 ± 20.7
GPT	30.4 ±3.2	32.7 ±3.9	25.8 ± 3.2	31.5 ±4.2	29 ± 3.6	28.5 ± 3.5

Data are expressed as the mean ± SE.

## Abbreviations

AP-1, activator protein-1; aSMase, acid sphingomyelinase; AU, arbitrary unit; BCA, bicinchoninic acid assay; DCF-DA, dichlorodihydrofluorescein diacetate; DMSO, dimethyl sulfoxide; ELISA, enzyme-linked immunosorbent assay; ECM, extracellular matrix; GOT, glutamic oxaloacetic transaminase; GPT, glutamic pyruvic transaminase; HBSS, hanks balanced salt solution; HRP, horseradish peroxidase; HDF, human dermal fibroblast; LAMP, lysosomal associated membrane protein; MMPs, matrix metalloproteinases; NF-κB, nuclear factor-κB; PVDF, polyvinylidene fluoride; PI, propidium iodide; ROS, reactive oxygen species; TWEL, transepidermal water loss; UVA, ultraviolet A; VEGF, vascular endothelial growth factor; ZLE, enzyme-treated *Zizania latifolia* extract
